# PHLPP is neither a Phosphatase nor a Tumor Suppressor

**DOI:** 10.1002/bies.70165

**Published:** 2026-07-12

**Authors:** Thomas A. Leonard

**Affiliations:** ^1^ Max Perutz Labs Medical University of Vienna Vienna Austria

**Keywords:** Akt signalling, cancer, cell biology, kinase, membrane, PHLPP, phosphatase, phylogenetics, Protein Kinase B, tumor suppressor gene

## Abstract

PH domain leucine‐rich repeat protein phosphatases 1 and 2 (PHLPP1/PHLPP2) have, for more than 20 years, been described as Akt phosphatases and tumor suppressor genes. Approximately 200 PubMed‐indexed articles have been published on the topic, including efforts to pharmacologically inhibit PHLPP in disease settings. A recent study, however, presents evidence that PHLPP is actually a pseudophosphatase with no catalytic activity. These observations question its reported role in cancer. The designation of PHLPP1 and PHLPP2 as pseudophosphatases necessitates both a critical examination of the literature and new hypotheses for the biological function of these ancient proteins. Here, we review the emergence of PHLPP1 and PHLPP2 as cellular signal transducers, critically examine the evidence for their reported phosphatase activity and tumor suppressor functions, and propose steps to elucidate their biological functions.

## Graphical abstract

PH domain leucine‐rich repeat protein phosphatases (PHLPP) have been described as Akt phosphatases and tumor suppressors for more than 20 years. Recent evidence, however, indicates that they are pseudophosphatases that lost catalytic activity more than 450 Mya. This article argues that new hypotheses are urgently needed to reorient the field.

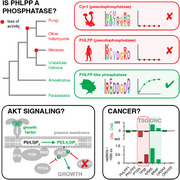

## 1 PHLPP Belongs to the PP2C Protein Phosphatase family

**FIGURE 1:**
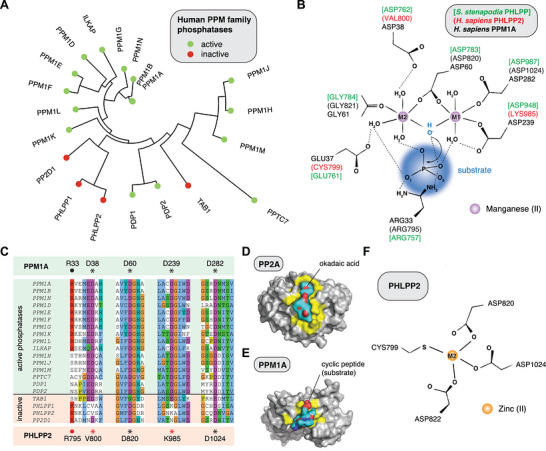
(A) Phylogenetic tree of human PPM family phosphatases. The tree was inferred using maximum likelihood in IQ‐TREE2 with automatic selection of the best‐fit amino acid substitution model (ModelFinder) and 1000 ultrafast bootstrap replicates for branch support. The tree was visualized in radial format using ggtree and branch lengths rescaled to improve separation of closely related proteins. The underlying structure‐based multiple sequence alignment was created using MUSTANG [162]. Active phosphatases (green), inactive pseudophosphatases (red). (B) Mechanism of PP2C‐mediated dephosphorylation. Figure adapted from Das et al. [4]. Metal‐coordinating residues mutated in PHLPP2 (red), metal coordinating residues in *S. stenapodia* PHLPP (green). (C) Multiple sequence alignment of metal‐ion coordinating and catalytic residues of human PPMs. (D) Structure of PP2A bound to okadaic acid (PDB: 2IE4). Okadaic acid binding pocket (yellow). (E) Structure of PPM1A bound to a cyclic peptide (PDB: 6B67). Peptide binding pocket (yellow). (F) Proposed metal ion center of human PHLPP2.

While phosphorylation of serine, threonine, and tyrosine residues in eukaryotes is accomplished by a single kinase domain fold which has been duplicated, adapted, and re‐used during the course of evolution [1], there are ten families of structurally distinct protein folds capable of performing the dephosphorylation reaction [2]. PH domain leucine‐rich repeat protein phosphatases 1 and 2 (PHLPP1 and PHLPP2) belong to the metal‐dependent family of protein phosphatases (PPM) also known as the PP2C (protein phosphatase 2 subtype C) family on account of their historical classification (Figure 1A). Widespread across all three kingdoms of life, PP2C phosphatases are associated with stress signaling, immunity, and development [3]. Catalysis of the dephosphorylation reaction is explicitly dependent on at least two divalent metal ions (widely believed to be manganese or iron) which serve to position and polarize the water molecule that attacks the phosphoester bond in a S_N_2 nucleophilic substitution reaction [4] (Figure 1B). Coordination of a third metal ion has been implicated in both catalytic activity and substrate specificity [5, 6, 7]. Mutation of any of the metal‐coordinating residues (Figure 1B, C), as well as a conserved arginine that coordinates the phosphate group of the substrate is sufficient to abrogate activity [7, 8, 9]. As such, active metallophosphatases (Figure 1A, green) are distinguished from their pseudoenzyme counterparts (Figure 1A, red) by the conservation of these residues. Conversely, pseudophosphatases, such as the PP2C family member TAB1 [10] are characterized by the mutation or absence of one or more of these motifs. Although formally classified bioinformatically as a pseudophosphatase in 2017 [2], PHLPP was known to encode residues incompatible with the coordination of metal ions essential for catalysis at the time of its discovery [11].

### 1.1 Vertebrate PHLPP Is a Pseudophosphatase

A recent study of human PHLPP2 found no evidence for phosphatase activity [12]. The authors discovered that low levels of phosphatase activity in purified PHLPP2 could be inhibited by the PP2A inhibitor okadaic acid and that trace amounts of all three subunits of the host expression system PP2A were present in preparations of the recombinant protein. Okadaic acid is specific for PP2A family phosphatases, including PP4 and PP6 [13] (Figure 1D). Even at concentrations of 10 µM, okadaic acid does not inhibit PP2C phosphatases [13], which are structurally unrelated to the PP2A family [2] (Figure 1E), and for which no selective inhibitors currently exist. When contaminating PP2A was removed by affinity‐purification using microcystin‐coupled phosphatase inhibitor beads [14], all residual catalytic activity was gone. The same effect could be observed in immunoprecipitates of PHLPP2 heterologously expressed in HEK293 cells, where serial washing of the beads also removed contaminating PP2A activity in the absence of okadaic acid. The authors subsequently discovered that PHLPP2 binds a single zinc ion in its catalytic site (Figure 1F), providing an explanation for the absence of catalytic activity. In contrast, ancestral‐like PHLPP proteins from Amoebozoa, which encode a complete active site (Figure 1B, green), exhibit robust metal‐dependent and okadaic acid‐insensitive phosphatase activity in vitro under the same conditions [12]. Experimental reports of PHLPP phosphatase activity are therefore deserving of special scrutiny. This brings us to reports that PHLPP1 and PHLPP2 are Akt phosphatases and, consequently, tumor suppressor genes.

### 1.2 Akt Signaling and Cancer

The serine/threonine protein kinase Akt is a key regulator of cellular and organismal growth and metabolism. Activation of receptors in the plasma membrane by growth factors and signaling molecules leads to the recruitment and activation of the lipid kinase phosphatidylinositol 3‐kinase (PI3K), which converts the bulk phosphoinositide phosphatidylinositol‐4,5‐bisphosphate (PIP_2_) into the lipid second messenger phosphatidylinositol‐3,4,5‐trisphosphate (PIP_3_). PIP_3_ elicits the specific recruitment, allosteric activation, and phosphorylation of Akt at the membrane [15, 16, 17], resulting in glucose uptake, inhibition of gluconeogenesis, upregulation of translation, and inhibition of apoptosis [18]. Dephosphorylation of PIP_3_ by the lipid phosphatase and tensin homolog (PTEN) serves to attenuate Akt signaling [19]. Deletion of one or two of three mammalian Akt genes results in reduced body weight and impaired glucose homeostasis [20], impaired brain development [21] or embryonic/neonatal lethality [22]. Conversely, Akt is hyperactivated in the majority of human cancers as a consequence of oncogenic mutations in RAS or the catalytic subunit of PI3Kα, or loss of the PTEN tumor suppressor gene [23]. A point mutation in Akt itself is causative of the mosaic overgrowth disorder proteus syndrome [24], as well as being associated with a spectrum of human cancers [25, 26, 27, 28].

The mechanisms by which PIP_3_ controls the spatial and temporal activity of Akt have been reviewed extensively elsewhere [29]. Briefly, the allosteric activation of Akt by PIP_3_ [15, 16, 17] licenses its phosphorylation on T308 in its activation loop by phosphoinositide‐dependent kinase 1 (PDK1) [30, 31] and S473 in its hydrophobic motif by mechanistic target of rapamycin complex 2 (mTORC2) [32]. These events serve to organize the catalytic machinery for phospho‐transfer [33, 34] and PIP_3_ confines Akt signaling to the membrane‐proximal environment [15]. ATP, as well as small molecule ATP‐competitive inhibitors, confer resistance to Akt dephosphorylation by phosphatases [35, 36, 37], a phenomenon which is dependent on PIP_3_ binding to its PH domain [16]. It follows, therefore, that Akt dephosphorylation is rate‐limited by its dissociation from the plasma membrane [15]. The PP2A family of protein phosphatases has long been implicated in the dephosphorylation of Akt T308, with both B55α [38] and B56β [12, 39] regulatory subunits proposed as mediators of specificity. The identity of the phosphatase that mediates hydrophobic motif (S473) dephosphorylation, however, is enigmatic.

### 1.3 Search for the Hydrophobic Motif Phosphatase

Motivated by a gap in our understanding of Akt dephosphorylation and signal termination, Gao et al. sought to identify a phosphatase specific for the hydrophobic motif of Akt [11]. Reasoning that Akt phosphorylation is serum‐sensitive, the authors searched the human genome for a phosphatase containing a PH domain. Having cloned the gene, the authors named it based on its domain architecture: PHLPP. PHLPP also contains, in addition to its PH and PP2C domains, an N‐terminal Ras‐associated (RA) domain. In the course of their investigation, the authors showed that PHLPP dephosphorylated Akt in vitro and in vivo, that overexpression of PHLPP in cancer cells induced specific dephosphorylation of Akt S473 and triggered apoptosis, and that knockdown of PHLPP prevented apoptosis in the same cells. They extended this analysis to show that downregulation of PHLPP led to increased Akt hydrophobic motif phosphorylation in *Drosophila* S2 cells. Finally, using human glioblastoma cells, they showed that stable over‐expression of PHLPP resulted in a reduction in tumorigenicity in mice xenografts. In 2007, the same group identified a paralog of PHLPP, PHLPP2, reporting its ability to dephosphorylate Akt, inhibit cell cycle progression, and induce apoptosis [40]. Since the identification of PHLPP1 and PHLPP2 as Akt phosphatases, hypothesis‐driven research has largely been viewed through the prism of cancer signaling. Where research has deviated from this perspective, it has also been built on the assumption that PHLPP is a bona fide protein phosphatase.

Before tackling the thorny question of whether PHLPP is a phosphatase or not, it is important to clarify the difference between direct, indirect, and correlative evidence. It is also crucial to differentiate biological effects from catalytic activity. There are many publications that have reported correlations between PHLPP and Akt phosphorylation in a multitude of different experimental settings, from overexpression to knockdown and knockout backgrounds and in genetically modified cells and organisms. However, since these studies do not establish direct causality or dependence on catalytic activity, they are not useful in answering the question of whether PHLPP is an active phosphatase. To answer this question, we must examine both biochemical and cell biological reports of PHLPP activity which include rigorous controls that specifically target catalytic activity.

## 2 Revisiting the Primary Data

Husremović and Meier et al. could not detect any phosphatase activity of purified recombinant PHLPP2 in vitro against full‐length Akt, phosphopeptides corresponding to T308 and S473 of Akt1, or the generic substrate *para*‐nitrophenylphosphate [12]. How can one reconcile these observations with previous reports of activity? The original report of PHLPP catalytic activity employed a GST‐PP2C fusion construct of PHLPP expressed in bacteria and purified in a single affinity step using glutathione Sepharose followed by elution with glutathione. Although *E. coli* does not encode a PP2A homolog, it does express an active PP2C family phosphatase [41]. Substrate Akt1 was expressed in baculovirus‐infected insect cells as a His_6_‐tagged protein and purified with a single NiNTA affinity step [11]. Although described as pure, no evidence of the purity or monodispersity of either protein was presented in the manuscript and, in vitro, okadaic acid was not employed to control for contaminating PP2A, a known Akt phosphatase. In addition, while millimolar concentrations of manganese were included in the reaction as a putative co‐factor for PHLPP, the authors did not demonstrate that manganese alone did not stimulate contaminating phosphatases in the purified Akt1 substrate. In fact, when this experiment was repeated by Husremović et al. using stoichiometrically phosphorylated Akt1 (purified by affinity, ion‐exchange, and size‐exclusion chromatography) [17] and de‐contaminated PHLPP2, there was no detectable dephosphorylation of Akt1 T308 or S473 [12].

The remainder of the Gao et al. study concerned itself with a combination of over‐expression and knockdown experiments, the interpretation of which was based on the understanding that the effects on Akt phosphorylation could be rationalized in the phosphatase activity of PHLPP. Although over‐expression of wild‐type PHLPP resulted in a modest ∼50% reduction in Akt S473 phosphorylation, deletion of the PH domain of PHLPP, which would be expected to impair its membrane association, resulted, paradoxically, in the same decrease in Akt S473 phosphorylation [11]. Conversely, deletion of the C‐terminal PDZ ligand of PHLPP abrogated the reduction in S473 phosphorylation conferred by wild‐type PHLPP, indicating that the PDZ ligand, but not the PH domain is necessary for Akt dephosphorylation. These observations were never reconciled with the authors’ working model in which the PH domain of PHLPP mediates its recruitment to the plasma membrane for the purpose of dephosphorylating Akt.

Almost 10 years after publication of the initial report identifying PHLPP1 as an Akt phosphatase, an in‐depth biochemical characterization of PHLPP1 and PHLPP2 was published [42]. Since PHLPP1 had previously been characterized as a hydrophobic motif phosphatase, the authors restricted their investigation to dephosphorylation of a pS473 peptide. The proteins were purified according to the protocols reported in their initial publication and were judged to be >90% pure by Coomassie‐stained SDS‐PAGE. Extremely low catalytic activities were detected and, again, okadaic acid was not added to exclude contamination with PP2A. While addition of manganese to PHLPP purified from either insect or mammalian cells increased catalytic activity, it had the opposite effect on the PP2C domain purified from bacteria. This implies that either the activity being measured did not come from PHLPP or that the proteins isolated from different expression hosts were not the same. Given that the contaminating PP2A found by Husremović et al. is both manganese‐dependent and common to insect and mammalian cells, there is a high likelihood that the activity reported was from a contaminant.

Finally, it is worth noting that at least two subsequent studies have concluded that both PHLPP1 [43] and PHLPP2 [44] elicit phosphatase‐independent effects. Although Madigan et al. [43] did not explicitly demonstrate an absence of catalytic activity in PHLPP1, the authors showed that, paradoxically, Akt and PHLPP1 had the same stabilizing effect on TBC1D7 and that the effect was independent of TBC1D7 phosphorylation by Akt. The authors also reported that, during the course of their study, they were unable to demonstrate catalytic activity of recombinant PHLPP1 PP2C domain. However, it should be noted that no primary supporting evidence for this statement was published. Agarwal et al. [44] observed that IKKβ phosphorylation was attenuated by wild‐type PHLPP2, but also by a phosphatase inactive mutant missing the phosphate‐coordinating arginine, leading them to conclude that the effect of PHLPP2 on IKKβ phosphorylation was independent of catalytic activity.

**FIGURE 2:**
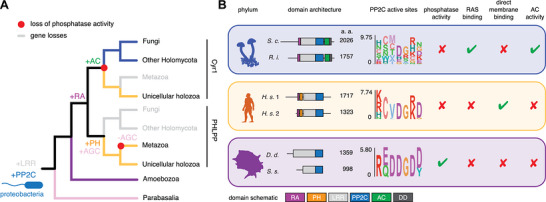
PHLPP is descended from an ancient gene formed approximately 1.2 billion years ago. In modern day amoeba and parabasalids, this PHLPP‐like phosphatase has an unknown function. Originally a phosphatase, PHLPP lost phosphatase activity at the base of the metazoan lineage. In fungi, an ancient gene duplication gave rise to CYR1, the major fungal adenylate cyclase. Essential for growth in yeast, CYR1 is part of the RAS‐CYR1‐PKA signaling pathway that controls hyphae formation via cAMP signaling and reorganization of the actin cytoskeleton. [RA (RAS‐associated domain); PH (Pleckstrin homology domain); LRR (Leucine‐rich repeat domain); PP2C (protein phosphatase 2C domain); AC (adenylate cyclase domain); DD (dimerization domain)].

In summary, there is no compelling direct evidence for phosphatase activity of either PHLPP1 or PHLPP2. The metal ion composition and stoichiometry of its catalytic site are incompatible with the known catalytic mechanism of PP2C phosphatases. In contrast, phylogenetics has revealed that PHLPP1 and PHLPP2 are derived from an ancient phosphatase that lost activity at the base of the metazoan lineage [12] (Figure 2). Gene duplication at the base of vertebrate evolution, after the loss of phosphatase activity, gave rise to two paralogous pseudoenzymes. An amoebozoan PHLPP ortholog that has retained a conserved catalytic site over more than one billion years (and was purified under the same conditions as human PHLPP2) exhibits robust, manganese‐dependent activity in vitro and is predictably insensitive to okadaic acid. Together, these observations indicate that PHLPP1 and PHLPP2 are most likely pseudophosphatases and that previous reports of catalytic activity were erroneously attributed to PHLPP.

### 2.1 Physiological Role of PHLPP in Akt Signaling?

This brings us to the question of whether PHLPP has any physiological role in Akt signaling, either indirectly or in a noncatalytic manner. The effects of PHLPP over‐expression on Akt phosphorylation are particularly difficult to deconvolute from potential pleiotropic effects on membrane signaling, trafficking or remodeling. This is due to the presence of a PH domain and C‐terminal PDZ ligand in PHLPP, both of which mediate its specific localization via protein‐lipid and protein‐protein interactions respectively. The PH domain binds to phosphoinositides in vitro [12] and bears significant similarity to the phosphoinositide‐binding PH domains of ArhGAP9 [45] and PLEKHA7 [46], while there are 23 other human proteins that share the same PDZ ligand. Endogenous PHLPP1 and PHLPP2 are expressed in HEK293 cells at concentrations of 1–9 nM as estimated by mass spectrometry [47]. Nevertheless, to study a potential effect of PHLPP on Akt signaling, cancer cell proliferation, or the dephosphorylation of other proteins in the cell, various investigators have overexpressed PHLPP [11, 40, 44, 48, 49, 50]; however, the magnitude of overexpression was not determined in these studies. Given that cellular membranes scaffold a diverse array of cellular processes, including vesicular transport, signal transduction, and cell morphogenesis [51], the overexpression of membrane‐binding proteins has significant potential to interfere with normal cellular physiology.

Next, let us consider genetic knockdown of PHLPP1 or PHLPP2. Whilst knockdown experiments do not suffer from the same pleiotropic effects that can be elicited by overexpression, they have their own limitations. Both off‐target effects and incomplete knockdown can affect the outcome of these experiments. Nevertheless, they can be a very useful tool with which to establish the role of a protein in a biological process. In the original report on PHLPP1, siRNA‐mediated knockdown of PHLPP1 in H157 cells (a human non‐small cell lung carcinoma cell line) resulted in increased S473 phosphorylation [11]. Knockdown of *Drosophila* PHLPP (dPHLPP) was also reported to affect dAkt S505 phosphorylation (S505 is the hydrophobic motif of dAkt) equivalent to that of dPTEN knockdown, though no evidence of either knockdown is reported, presumably due to the absence of antibodies that could detect either dPTEN or dPHLPP. Subsequent studies have linked PHLPP1 and PHLPP2 knockdown to Akt isoform specificity [40], increased Akt phosphorylation and tumorigenesis [44, 49, 52], and cell proliferation [44, 53]. However, in a separate study, knockdown of PHLPP1 in LN229 cells did not elevate Akt S473 phosphorylation [54] and knockdown of PHLPP2 in two subsequent studies revealed no significant effect on Akt S473 phosphorylation in 3T3‐L1 adipocytes [55] or PTEN^Δ/Δ^ TRP53^Δ/Δ^ mouse embryonic fibroblasts (MEFs) [56]. Overexpression of PHLPP2 in the same MEFs also had no effect on S473 phosphorylation [56]. Although no consistent pattern of reduced Akt phosphorylation upon PHLPP knockdown in different cell lines has been established, we cannot exclude the possibility that PHLPP acts in a cell context‐dependent manner.

Finally, the original report showed that four cancer cell lines (two colorectal adenocarcinoma cell lines – DLD1, HT29; two glioblastoma‐derived cell lines – LN‐319, LN444) exhibited differential Akt phosphorylation which correlated with their respective PHLPP expression levels [11]. Notably, however, T308 and S473 phosphorylation are coupled, indicating that the correlation is not specific to S473. Furthermore, control data referenced in the text, including relative expression levels of PTEN, are not shown (data not shown). Ultimately, these observations represent a small vignette of three selected markers in a genotypically diverse set of cancer cell lines. Overexpression of PHLPP in two of the four cell lines showed a modest effect on S473 phosphorylation, but expression of a PHLPP mutant lacking the PP2C domain was not included in the controls. When PHLPP was stably expressed in LN229 glioblastoma cells, both T308 and S473 phosphorylation of Akt were reduced compared to a control vector, but tumors derived from the two independent nude mice injected with PHLPP‐transfected LN229 cells exhibited no difference in Akt S473 phosphorylation despite orders of magnitude different PHLPP expression levels. The conclusion that PHLPP suppresses tumorigenicity via Akt S473 dephosphorylation is therefore difficult to reconcile with the 68% reduction in tumor volume observed in the four mice injected with PHLPP1‐expressing LN229 cells. These observations question whether the reported tumor suppressor activity can be reasonably attributed to PHLPP phosphatase activity.

### 2.2 Evidence of a Tumor Suppressive Function

In this light, then, let us examine the evidence that PHLPP1 and PHLPP2 are tumor suppressors, irrespective of their precise structural or biochemical role.

The catalog of somatic mutations in human cancer (COSMIC) database reveals no hotspot mutations in either PHLPP1 or PHLPP2 [12, 57]. The mutation rates of both genes are comparable to two olfactory receptor genes chosen at random and reflect background mutation rates unrelated to tumorigenesis. In contrast to bona fide oncogenes or tumor suppressors, neither PHLPP1 nor PHLPP2 genes are gained or lost (copy number variants) at rates significantly different from olfactory genes. Numerous cancer dependency studies have shown that neither PHLPP1 nor PHLPP2 are essential in more than 1000 cancer cell lines [58, 59, 60, 61, 62, 63, 64, 65], and no synthetic lethal interactions involving either gene have been found in cancer cells [66]. Finally, genome‐wide association studies (GWAS) have not revealed any cancer associations for the genetic loci of PHLPP1 or PHLPP2. In summary, there exists no circumstantial evidence that might indicate a link between cancer and PHLPP1 or PHLPP2.

Tumor suppressor genes have been widely characterized by examining the phenotypes of mice in which one or both alleles have been knocked out. For example, both homozygous and heterozygous mice with germline deletions of p53 are developmentally normal, but susceptible to spontaneous tumor development [67, 68]. Deletion of the retinoblastoma gene in mice leads to embryonic lethality [69], while heterozygous mice have a propensity to develop tumors arising from cells in which the wild‐type allele is absent [70]. Similarly, PTEN is required for embryonic development and haploinsufficient *pten^+/−^* mice exhibit hyperplasia in the prostate, skin and colon [71] characteristic of three human autosomal dominant disorders associated with PTEN germline mutations: Cowden disease (CD), Lhermitte‐Duclos disease (LDD), and Bannayan‐Zonana syndrome (BZS), collectively known as PTEN hamartoma tumor syndromes (PHTS) [72]. Loss‐of‐heterozygosity (LOH) is frequently observed in tumors of *pten^+/−^* mice [73]. Knockout PHLPP1 and PHLPP2 mice, as well as a double knockout mouse have also been generated [74, 75, 76], but all are viable and none exhibit any gross developmental or anatomical defects.

Although an increased propensity of PHLPP1 or PHLPP2 knockout mice to develop invasive tumors has not been reported, high‐grade prostatic intraepithelial neoplasia has been reported in the prostates of 8‐month‐old *phlpp1^−/−^* mice [77]. Both PHLPP1 and PHLPP2 have also been reported to potentiate PTEN‐mutant prostate cancer progression [56, 77, 78]. Genetic studies in mice have shown that neoplasia and cancer in PTEN‐deficient prostate cells depend specifically on Akt S473 phosphorylation by mTORC2 [79]. Based on the reports of S473 dephosphorylation by PHLPP1 and PHLPP2, cancer scientists have since used mouse models and patient whole genome analyses to probe the relevance of PHLPP in the progression of prostate cancer from indolent to aggressive disease. Mice heterozygous for *pten* [71] were crossed with the *phlpp1* null mice generated by Masubuchi [76], generating six different genotypes [77]. Curiously, *phlpp1^−/−^* mice wild‐type for *pten* exhibited a significant reduction in lifespan compared to both wild‐type and heterozygous *phlpp1^+/−^* littermates [77]. Mice heterozygous for *pten* exhibited a significant reduction in survival which was further reduced by the loss of both *phlpp1* alleles, suggestive of a synergistic effect. However, though S473 phosphorylation was observed to be elevated in both *phlpp1^−/−^* and *pten^+/−^ phlpp1^−/−^* animals, foci of activated Akt observed by immunohistochemistry were typically associated with reduced PTEN levels. In light of the recent finding that PHLPP is a pseudophosphatase, it is more likely that changes in Akt phosphorylation in these tissues were the consequence of variable PTEN expression and/or activation rather than a direct consequence of the loss of PHLPP1. Inactivating mutations in p53, elevated in *pten^+/−^ phlpp1^−/−^* prostates compared to the wild‐type and *pten^+/−^* controls, likely contributed to the emergence of neoplastic disease. Why sporadic p53 mutations arose specifically in the *pten^+/−^ phlpp1^−/−^* mice and whether there is a causal link is unclear. Although *phlpp1* gene loss has been observed in a small subset of primary and metastatic prostate cancers, 76% of cases also involved co‐deletion of the known tumor suppressor gene *smad4* [80, 81], mutations of which are a leading cause of juvenile polyposis syndrome [82]. 18q21 Deletion Syndrome, which covers the genomic locus of *phlpp1*, results in the co‐deletion of *smad2, smad4, smad7, bcl2*, and *dcc*, all of which are curated cancer genes [81].

PHLPP2 has similarly been implicated in prostate cancer progression, but via an entirely different mechanism to PHLPP1. Using a mouse model of PTEN^Δ/Δ^, TP53^Δ/Δ^ metastatic prostate cancer, the group that identified a role for PHLPP1 in prostate cancer also found that reduced Akt S473 phosphorylation was associated with elevated PHLPP2 levels and interleukin‐6 (IL6)‐driven activation of the MYC oncogene [78]. Deletion of PHLPP2 in a follow‐up study was found to block tumor growth via effects on the stability of MYC [56]. Mechanistically, the PHLPP2‐mediated dephosphorylation of T58 of MYC was proposed to stabilize MYC, thereby potentiating MYC‐mediated proliferation. Phosphorylation of T58 is required for PP2A‐mediated dephosphorylation of S62 and subsequent degradation by the ubiquitin‐proteasome system [83], such that dephosphorylation of T58 would promote maintenance of a stable, T62‐phosphorylated pool with oncogenic potential. However, given recent evidence that PHLPP2 is a pseudophosphatase, other genetic alterations such as MYC amplification may have contributed to metastasis. Consistent with this hypothesis, tandem duplication hotspots near MYC have been identified as a common hallmark of metastatic prostate cancer [84].

Both PHLPP1 and PHLPP2 have also been associated with colon cancer progression. Using immunohistochemical staining with isoform‐specific antibodies against PHLPP1 and PHLPP2, one study reported the loss of PHLPP expression in colorectal cancer cells and that proliferation of colorectal cancer cells could be inhibited by PHLPP1 overexpression [49]. However, the PHLPP1 antibody used was later found to cross‐react strongly with the proto‐oncogene β‐catenin [85] an observation subsequently corroborated by the antibody manufacturer, with the product withdrawn from sale [86].

A number of other studies have reported correlations between PHLPP1 or PHLPP2 loss and various cancers, with a majority citing attenuated dephosphorylation of Akt as the causal factor [49, 87, 88, 89, 90, 91]. While PHLPP has been linked to PTEN signaling in glioblastoma via the scaffold protein Na(+)/H(+) exchanger regulatory factor 1 (NHERF1), silencing of PHLPP1 had no effect on Akt S473 in the absence of PTEN silencing [54], while expression of a putatively phosphatase‐inactive mutant of PHLPP2 in a follow‐up study from the same lab led the authors to conclude that PHLPP2 exerts noncatalytic effects on NF‐κB signaling [44]. In summary, the links between PHLPP and cancer are based on correlative observations and the attribution of phosphatase activity to PHLPP‐mediated regulation of Akt signaling. While a role in cancer cannot be entirely dismissed, neither PHLPP1 nor PHLPP2 qualifies as a high‐priority target on the balance of the evidence available.

### 2.3 Implication of PHLPP in Other Cellular Processes

In addition to Akt signaling, PHLPP has been implicated in PKC signaling [48], tyrosine kinase signaling [92], cardiac hypertrophy [93, 94], control of the circadian clock [76], cell cycle regulation [40], bone morphogenesis [95, 96, 97], diabetes [98], colitis [74], mitosis [99], obesity [55], and colorectal cancer development [100]. Histones [92], GRK5 [93], MST1 [98], hormone‐sensitive lipase (HSL) [55], RAF1 [100], and S6K1 [50] have all been proposed to be PHLPP substrates. Each of these studies has linked phosphatase activity of PHLPP to its biological function as a negative regulator of kinase signaling.

Phosphorylation of the hydrophobic motif is required for the proper folding of PKC and maintenance of its autoinhibited conformation in cells [101]. Dephosphorylation by a PP2A phosphatase leads to rapid PKC degradation [102]. PHLPP1 has been implicated in the dephosphorylation of the hydrophobic motifs of PKCα and PKCβ [48] and their subsequent degradation [103]. However, ectopic expression of PHLPP1 in cells had no effect on PKCβII phosphorylation kinetics. Somewhat paradoxically, both PHLPP1 and PKC have been described as tumor suppressors [104, 105], despite the reported role of PHLPP1 in opposing both Akt and PKC function [11, 40, 48]. Of the ten mammalian PKC genes, only PRKCB is classified as a cancer gene (Tier 2) in the Cancer Gene Census [81]. Irrespective of whether PKC has a role in cancer, however, its (dys)regulation by PHLPP1 or PHLPP2 is at best indirect.

PHLPP1 knockout mice have been reported to have an impaired capacity to reset their circadian rhythms [76] and to exhibit protection from ischemic brain injury [75]. Although *phlpp1* ^−/−^^−/−^ mice were determined to exhibit circadian rhythm differences from their littermate controls, the study did not determine any mechanistic link to Akt signaling [76]. Conversely, Akt signaling in *phlpp1* ^−/−^ mice was specifically investigated in response to middle cerebral artery occlusion, with rather modest effects on infarct volume and Akt S473 phosphorylation [75]. PHLPP double knockout mice have been reported to exhibit an inflammatory bowel (colitis) phenotype, although the mice are viable and develop normally [74]. The study reports correlations with PHLPP expression levels in five human inflammatory bowel disease (IBD) patients which are highly variable and do not systematically correlate with Akt S473 phosphorylation. None of these studies definitively links PHLPP phosphatase activity to the phenotype observed. It is therefore eminently plausible that PHLPP loss contributed to the phenotypes as a catalytically inactive pseudoenzyme.

### 2.4 Pharmacology of a Pseudophosphatase

The designation of PHLPP1 and PHLPP2 as tumor suppressors does not logically lead to therapeutic applications in oncology, since it is a loss of function, putatively, that has been attributed to the disease phenotype. Nevertheless, therapeutic avenues have been identified in the treatment of metastatic prostate cancer [56, 77], cardiac arrest survival [106], heart disease and diabetes [107]. In the case of metastatic prostate cancer, the logic of PHLPP2 inhibition stemmed from the idea that preventing T58 dephosphorylation of MYC would promote its PP2A‐mediated degradation [56]. Chemical and in silico screening of a small molecule repository of the National Cancer Institute (NCI) has claimed to identify inhibitors of PHLPP phosphatase activity in vitro, with a corresponding increase in basal and agonist‐elicited Akt phosphorylation in cells [107]. The screen was performed with the PP2C domain of PHLPP2 recombinantly expressed in bacteria. Details of the purification protocol and analysis of the purity of the resulting protein were not reported in the manuscript. A set of 20 hit compounds was identified with IC_50_ values less than 100 µM and that reduced the signal in the assay by more than 50%. Cells were treated with 10–250 µM of the six most promising compounds for 24 h to assess their effects on Akt S473 phosphorylation. Of the six, two compounds (NCS117079 and NCI45586) were further evaluated for their inhibitory potential in vitro. Coincidentally, despite having considerably different structures, these compounds exhibited near identical IC_50_ values against the PP2C domain of PHLPP2. Given that the bacterially‐expressed PP2C domain of PHLPP2 was later reported by the same authors to have 100‐fold lower activity than the same domain purified from insect cells [42] and recent attribution of the activity in PHLPP2 preparations from insect cells to contaminating PP2A [12], it is not clear what activity the authors were measuring in their assay. Nevertheless, NCI45586, the most potent compound identified in the study has subsequently been used to support claims that pharmacological inhibition of PHLPP could be a viable therapeutic avenue in prostate cancer treatment [56], the treatment of intervertebral disc degeneration associated with chronic back pain [108], cartilage regeneration in osteoarthritis [109, 110], and neuroprotection after ischemia [111]. NCI117079 has been used to support a role for PHLPP1 in chaperone‐mediated autophagy [112]. Intra‐articular injection of NCI117079 into post‐traumatic osteoarthritic joints of mice in which PHLPP1 was deleted from chondrocytes at 12 weeks after birth reduced PTOA‐induced cartilage damage [113], implying that the inhibitor exerts an effect either on PHLPP2 or, more likely, on another pathway. Determination of the biological targets of these compounds would therefore be helpful in rationalizing their observed effects.

### 2.5 PHLPP or SCOP?

Prior to 2005, PHLPP was known by a different name – Suprachiasmatic nucleus (SCN) circadian oscillatory protein (SCOP), on account of the circadian oscillation of transcripts of the gene in the SCN [114]. Subsequent studies linked SCOP to the downregulation of MAPK/ERK signaling [115] and memory formation in the hippocampus [116], albeit without any focus on the phosphatase domain. Although K‐RAS has been identified as a binding partner of the RA domain of SCOP [115], the surface of the RA domain involved in RAS binding has diverged considerably since the genetic acquisition of a PH domain. While in vertebrates, the RAS‐interaction surface of the RA domain of PHLPP is the least conserved surface on the protein [12], the fungal homolog of PHLPP, the adenylate cyclase (AC) Cyr1, depends crucially on RAS binding to drive membrane association and cAMP signaling [117, 118] and exhibits a correspondingly conserved surface predicted to bind RAS [12]. Nevertheless, interest in SCOP and utilization of the SCOP nomenclature was rapidly superseded by the new designation of PHLPP and its role in cancer. A plethora of review articles have since consolidated the proposed role of PHLPP in the attenuation of protein kinase signaling in a wide spectrum of physiological and pathological contexts [105, 119, 120, 121, 122, 123, 124, 125].

### 2.6 Insights From Phylogenetics and Pseudoenzymes

Investigation of the biological function of PHLPP will undoubtedly be helped by a fresh, unbiased approach. So, what do we know with high confidence? PHLPP1 and PHLPP2 are derived from an ancient gene that first appears in the evolutionary record over 1 billion years ago (Figure 2A). In its first incarnation, it was a genuine phosphatase with unknown substrates. Orthologs of this ancestral phosphatase persist in modern‐day amoebozoans and parabasalids with experimental evidence for metal‐dependent, okadaic acid‐insensitive phosphatase activity [12] (Figure 2B). In the common ancestor of opisthokonts (animals and fungi), the ancestral phosphatase acquired an RA domain and was duplicated, giving rise to the PHLPP and Cyr1 families. The PHLPP ancestor subsequently acquired its characteristic PH domain and an AGC kinase domain, whereas the Cyr1 family acquired an adenylate cyclase (AC) domain. The presence of both PHLPP and Cyr1 homologs in unicellular holozoans, such as filastereans and ichthyosporeans, traces this ancient duplication to the ancestor of opisthokonts, suggesting that PHLPP and Cyr1 genes were lost in fungi and holozoans, respectively. In the PHLPP family, phosphatase activity was lost at the onset of the metazoan lineage, possibly coincident with the loss of the kinase domain. Metazoans therefore encode PHLPP pseudophosphatases (Figure 2B). In fungi, Cyr1 is an essential component of the RAS‐AC‐PKA growth signaling pathway, despite also having lost phosphatase activity approximately 1 billion years ago (Figure 2A‐B). Studies of pseudoenzymes, however, should caution us against dismissing the possibility of non‐canonical catalytic activity. Pseudokinases with unexpected catalytic activities, including AMPylation [126] and glutamylation [127], mediated by the same kinase fold as a bona fide serine/threonine or tyrosine kinase, have been identified. Conversely, the *Legionella pneumophila* effector protein SidD uses a PP2C domain to de‐AMPylate human RAB1 during its intracellular infection cycle [128, 129]. In the realm of lipid phosphatases, which evolved from protein tyrosine phosphatases, six members of the myotubularin (MTM/MTMR) family are pseudophosphatases, having lost key catalytic residues. Nevertheless, they play important roles in scaffolding and regulating the activity of MTM phosphatases, with mutations in MTMR5 and MTMR13 causative of type 4B Charcot‐Marie‐Tooth disease [130, 131, 132].

**FIGURE 3:**
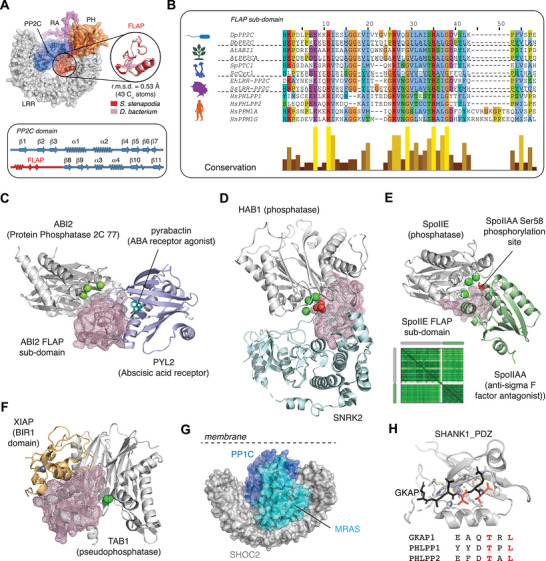
(A) PP2C phosphatases are characterized by an insertion between strands β7 and β8 of the phosphatase domain, called the FLAP domain. (B) The FLAP domain of PHLPP is found throughout all kingdoms of life. (C) Crystal structure of the ABA receptor PYL2 (light blue) in complex with pyrobactin and the Protein Phosphatase 2C 77 (ABI2, grey) of *A. thaliana*. The ABI2 FLAP domain is depicted in pink mesh; pyrobactin is depicted in sticks. (D) Crystal structure of the complex between the HAB1 phosphatases and SNRK2. A sulphate molecule from the crystallization solution corresponding to the position of the activation loop Ser175 is shown in spheres. The HAB1 FLAP domain is depicted in pink mesh. (E) AlphaFold model of the complex between *B. subtilis* SpoIIE (phosphatase, grey) and the antisigma F factor antagonist SpoIIAA (light green). The SpoIIE FLAP domain is depicted in pink mesh; the phosphorylation site of SpoIIAA, Ser58, is shown in red sticks. (F) Crystal structure of the TAB1 pseudophosphatase in complex with the BIR1 domain of XIAP. The TAB1 FLAP domain is depicted in pink mesh. G. Structure of SHOC2‐PP1C‐MRAS complex (PDB:7SD0) superimposed on the structure of PHLPP2 and shown in the same orientation as panel A. H. Structure of the SHANK1_PDZ‐GKAP1 complex (PDB: 1Q3P).

The PP2C domain itself has prokaryotic origins. Bacterial PP2C phosphatases play important roles in stress signaling, including the regulation of cell wall integrity [133, 134], sporulation [135], and carbon and nitrogen sensing [136]. The substrate specificity of both bacterial and eukaryotic PP2C family phosphatases is derived from a variable insertion in the phosphatase domain that creates the so‐called FLAP subdomain adjacent to the catalytic site [137, 138] (Figure 3A, B). In plants, PP2C phosphatases play critical roles in abscisic acid (ABA) signaling [139, 140, 141] (Figure 3C), including the inactivation of subfamily 2 Snfl‐related kinases (SnRK2s) [142] (Figure 3D). The bacterial sporulation factor SpoIIE is a PP2C phosphatase that dephosphorylates SpoIIAA to activate the transcription factor Sigma F [135] during asymmetric cell division (Figure 3E). Finally, the human pseudophosphatase TAB1 uses the opposite surface of its FLAP subdomain to bind to the BIR1 domain of X‐linked inhibitor of apoptosis (XIAP), an interaction which is critical for transforming growth factor beta‐activated kinase 1 (TAK1)‐mediated activation of NF‐κB signaling [143] (Figure 3F). Although vertebrate PHLPP likely lost catalytic activity, the FLAP domain, along with the PH domain, is the most highly conserved surface over more than 450 million years. These observations imply that human PHLPP1 and PHLPP2 may have lost phosphatase activity but retained binding to their ancestral substrates.

## 3 Biological Significance

That PHLPP lost phosphatase activity prior to the evolution of metazoans does not imply that it is any less important to animal biology. An evolutionary pressure of some sort has retained at least one PHLPP gene in all sequenced gnathostomes (jawed vertebrates). Interestingly, PHLPP has been entirely lost in cnidaria (e.g., anemones, corals, and jellyfish) for reasons that are currently not understood. Pseudoenzymes, including pseudokinases [144], pseudophosphatases [145], pseudoGTPases [146], and pseudoproteases [147, 148] are abundant in biological systems, with a molecular understanding of their function in many cases. A logical place to start, then, would be to characterize the subcellular localization and interactome of PHLPP. These efforts will undoubtedly be complicated by the low abundance of PHLPP1 and PHLPP2 in cells [47], for which high‐throughput tagging of the endogenous genes has so far failed to realize any subcellular localization or interactome data [149]. Instead, interaction partners of PHLPP have been identified in large scale affinity‐purification mass spectrometry (AP‐MS) screens [150, 151, 152], targeted AP‐MS studies [43, 153, 154, 155], proximity labeling [99], or targeted pull‐down experiments [54, 156]. Most of the targeted studies have described mechanisms that relate the interaction partners to Akt signaling, including Scribble [156], the deubiquitinases (DUBs) USP1 and USP12 [99, 155, 157, 158], and the DUB accessory factors WDR20 and WDR48 [99, 155, 157]. All of these interactions must be treated cautiously, however, given that they are derived from over‐expression of the bait protein and that the mechanisms proposed are predicated on the dephosphorylation of Akt by PHLPP. Interestingly, the closest structural homolog of the LRR domain of PHLPP is a protein called SHOC2. In a case of convergent evolution, SHOC2 forms a complex with the structurally unrelated phosphatase PP1C, which binds to the same surface of the LRR domain as the PP2C domain of PHLPP2. The SHOC2‐PP1C complex specifically binds MRAS within the LRR cradle of SHOC2 to mediate its dephosphorylation [159]. The structure (Figure 3G) illustrates the physical constraints of substrate binding that likely also apply to PHLPP. Finally, both PHLPP1 and PHLPP2 encode a PDZ ligand at their C‐termini with specificity for type Ic PDZ domains [160] (Figure 3H). Although the terminal DTXL motif is shared by 23 other human proteins and there are 15–30 type 1c PDZ domains encoded in the human proteome, PHLPP1 and PHLPP2 have been reported to interact with NHERF1 via its second PDZ domain (PDZ2) [54], which has a strong preference for ‐3 acidic residues [161]. The interactions of PHLPP1 with Scribble and PHLPP1 and PHLPP2 with NHERF1 have been validated by truncation of their respective C‐terminal PDZ motifs [54, 156].

Preliminary investigation of the membrane binding properties of PHLPP2 suggest that it has a preference for mono‐ and di‐phosphorylated phosphoinositides [12]. Whilst consistent with the known phosphoinositide binding of structurally related PH domains [45, 46], systematic determination of the lipid binding specificity of the PH domain using synthetic membranes is still required. Evidence of phosphoinositide binding, coupled to conservation of the PH domain, is, however, sufficient to indicate that PHLPP likely fulfills a scaffolding role within the endolysosomal membrane system. Immunofluorescence or tagging of the endogenous gene would undoubtedly shed much‐needed light on the subcellular compartment(s) to which PHLPP belongs. Ultimately, the subcellular localization of PHLPP should converge on the identification of compartment‐specific interaction partners.

## 4 Conclusions

New experimental evidence indicates that neither PHLPP1 nor PHLPP2 is a bona fide protein phosphatase. Neither PHLPP1 nor PHLPP2 qualifies as a cancer gene according to the strict criteria of the Cancer Gene Census. Evidence for their role in cancer is therefore limited to observations arising from the targeted analysis of specific markers in human cancer cell lines upon overexpression or knockdown of PHLPP. Given the aforementioned risk of pleiotropic effects that could arise from the overexpression of a membrane‐binding protein and the obvious off‐target effects of small molecule PHLPP inhibitors, much of this work should be treated with caution. However, genetic perturbation of PHLPP in multiple independent laboratories has implicated it in a variety of cellular processes and the evolutionary record tells us that it must be important. Elucidation of the precise biological function of these pseudophosphatases will require careful mapping of their subcellular localization and interactomes before any further mechanistic studies can be conducted.

## Conflicts of Interest

The authors declare no conflicts of interest.

## Data Availability

Data sharing not applicable to this article as no datasets were generated or analyzed during the current study.
